# *Xenopus* Interferon and Viral Mimics: Insights into Evolution and Adaptation of Immune Response in Vertebrates

**DOI:** 10.3390/cimb48080796

**Published:** 2026-08-06

**Authors:** Collins Khwatenge, Olayimika Adeyemi

**Affiliations:** Department of Biological Sciences, Delaware State University, Dover, DE 19901, USA

**Keywords:** interferons, viral mimics, immune evolution, JAK–STAT signaling, host–pathogen coevolution

## Abstract

The immune system is a critical system in evolutionary biology, shaped by coevolution between hosts and pathogens. The frog *Xenopus* is a valuable model organism for studying this process, offering insights into amphibian development, immunity, and the evolutionary dynamics of the interferon (IFN) response and viral interactions. The role of *Xenopus* IFN and the viral mimics introduced during the development of the immune system is discussed in this review, and the roles they played in the study of host–pathogen interactions and in the replication of genes that allowed the development and complexity of the immune system are reflected. We discussed the molecular mechanisms of *Xenopus* IFN signaling and the emergence of viral mimics in evolutionary biology. This review offers insight into how immune systems evolve under the pressure of pathogens, and the understanding of *Xenopus* IFN, its viral mimics, and that of other leading players in the vertebrate immune system may have further applications in medicine and biotechnology. *Xenopus* is a good model for studying the evolutionary dynamics of immune systems. In conclusion, this review posits that further elucidation of vertebrate immune adaptation mechanisms—particularly the evolutionary refinement of IFN-mediated antiviral restriction pathways, as demonstrated by *Xenopus* IFNs counteracting viral molecular mimics—will critically inform the rational design of next-generation antiviral pharmacotherapies, structure-guided vaccine adjuvants, and targeted immunomodulatory biologics.

## 1. Introduction

### 1.1. Overview of the Immune System and Evolution

The immune system is a dynamic and constantly evolving network, shaped by relentless selective pressure from diverse pathogens. This ongoing evolutionary arms race has driven the organization of host defense into two complementary branches: innate and adaptive immunity, both essential for host protection [1,2,3,4,5]. The innate immune system provides immediate, nonspecific defense, while the adaptive immune system mounts slower but highly specific responses, generating immunological memory. Over millions of years, hosts have evolved sophisticated defense mechanisms, while pathogens have reciprocally developed strategies to evade or subvert them. Consequently, immune genes and pathways have diversified through a coevolutionary struggle involving viruses, bacteria, fungi, and multicellular organisms [6,7].

A comprehensive understanding of this evolutionary trajectory requires examining key transitional species. In this context, amphibians—particularly the African clawed frog (*Xenopus*)—represent a critical phylogenetic bridge between fish and amniotes (reptiles, birds, and mammals). The *Xenopus* immune system exhibits a unique combination of ancestral and derived features: it possesses a fully functional adaptive immune system with classical major histocompatibility complex (MHC) class I and II molecules, T-cell receptors, and immunoglobulins, yet it also retains primitive characteristics, such as a greater reliance on innate-like lymphocytes and distinct IFN responses. Notably, *Xenopus* has served as an invaluable model for elucidating the developmental origins of the immune system, the evolution of the MHC, and the emergence of alternative adaptive immune mechanisms, such as those involving non-rearranging antigen receptors [8,9,10,11]. By analyzing such intermediate taxa, scientists gain deeper insights into how pathogen–host interactions have shaped immune architectures across vertebrate phylogeny [6,7].

At the individual level, the immune system exhibits short-term adaptability, enabling organisms to respond to novel threats during their lifetime through epigenetic regulation and transcriptional reprogramming [12,13,14]. At the population level, long-term evolutionary adaptability is driven by the polymorphism of immune genes, ensuring that within a population, some individuals carry advantageous variants that enable their immune systems to recognize and effectively counter new or mutated pathogens, thereby securing species survival [7,11]. Positive selection of variants that enhance pathogen recognition and effector functions promotes the diversification of immune genes. Rapid changes in immune responses are orchestrated by epigenetic modifications [12,13,14] and critical transcription factors, such as signal transducer and activator of transcription (STAT) 1 and STAT2 in the Janus kinase (JAK)-STAT signaling pathway, which form complexes to regulate interferon-stimulated genes (ISGs) and drive antiviral responses [15,16,17,18]. The combination of these mechanisms conditions the immune response to dynamically react to pathogen challenges.

**Figure 1:**
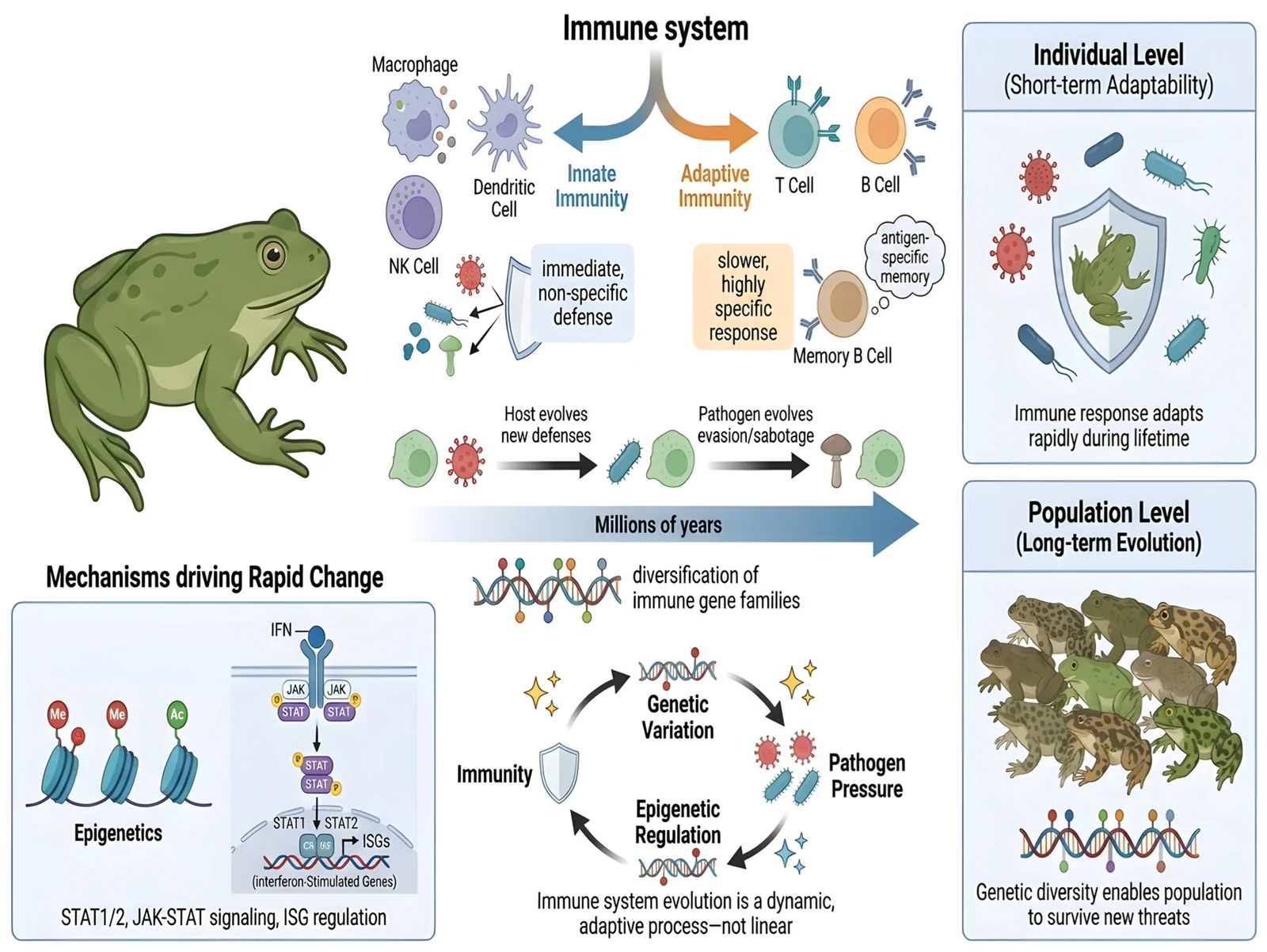
*Xenopus* immune system and its evolution: The *Xenopus* model at the center branches out depicting the two main arms of the immune system: innate immunity (with immediate and non-specific defense icons) and adaptive immunity (showing slower, highly specific responses and antigen-specific memory formation). It also includes a timeline representing the evolutionary arms race between *Xenopus* and pathogens like viruses, bacteria, and fungi, highlighting individual versus population-level immune adaptability through gene diversity, and illustrates the role of epigenetic regulation and the JAK-STAT pathway in controlling immune responses. The immune system evolution is an ongoing, dynamic, and non-linear process shaped by genetic, epigenetic and environmental forces. This figure was generated using the FigureLabs AI platform, version 2026 (https://chat.figurelabs.ai; accessed 29 May 2026) based on the authors’ original concept and instructions; the final image was reviewed and edited by the authors.

The need for rapid adaptation is particularly evident in the IFN family, which has undergone extensive gene duplication and functional diversification across vertebrates—a process elegantly illustrated in *Xenopus*, where multiple IFN subtypes have been identified, reflecting lineage-specific expansions driven by viral pressures [19,20,21]. In sum, the immune system does not develop in a linear or straightforward manner; rather, it is a dynamic and adaptive process shaped by evolutionary forces that govern an organism’s genetic makeup and continuously refine its immunological capabilities (Figure 1).

### 1.2. Xenopus as a Model for Immunological Research

African clawed frogs of the *Xenopus* genus, particularly the species *Xenopus laevis* and *Xenopus tropicalis,* whose genome has now been fully sequenced and annotated [6,22] are beneficial amphibian model organisms for studying vertebrate development because of their simple biology, the size of their embryos, and their ease of manipulation [23,24]. *Xenopus* is an excellent model for immunology [25] and evolution [26]. Due to its unique conditions, it has been of great significance to the evolutionary tree and is a valuable resource for understanding the vertebrate immune system. As a jawed vertebrate, *Xenopus* possesses an immune system remarkably similar to that of mammals, including B and T lymphocytes, major histocompatibility complex (MHC), and key cytokines. The phylogenetic position of *Xenopus* as an amphibian—intermediate between aquatic fish and terrestrial mammals—allows researchers to distinguish evolutionarily conserved immune mechanisms from species-specific adaptations [6,26,27].

*Xenopus* develops externally, free from maternal influence, providing easy experimental access from embryonic through larval stages. The tadpole’s transparency is a major advantage for visualizing processes like cell migration, tissue regeneration, and lymphatic development in real-time. Furthermore, the immune system undergoes a dramatic remodeling during metamorphosis, offering a unique opportunity to study immune differentiation and the establishment of self-tolerance [25,28].

The integration of novel loss-of-function techniques, advanced genome editing tools, and efficient transgenesis has established *Xenopus* as a powerful and versatile platform for immunological research [29]. The growing genetic resources and tools that allow for the manipulation of the genetic code have made it an even more useful model organism. Specifically, adjustments to CRISPR/Cas9 technology enable precise gene editing in *Xenopus*, facilitating detailed functional studies of immune-related genes [30,31,32]. By employing these technologies, researchers can determine how various genes play a role in the immune response function. Furthermore, the development of advanced transcriptomic and proteomic technologies has provided new insights into the development and immune mechanisms in *Xenopus* [33,34,35,36,37,38,39,40]. These new methods allow for the detailed study of gene expression and protein intermolecular interactions during immune processes, down to the individual molecular level. Ultimately, the unique attributes of *Xenopus* make it applicable to a wide range of immunological investigations, spanning basic biology to translational medicine (Table 1).

### 1.3. Interferons (IFNs) and Viral Mimics

Interferons are cytokines, one of which is perpetually involved in the antiviral response. They significantly contribute to defense against viral infections by inducing the expression of hundreds of ISGs and suppressing viral replication [51,52,53]. The interferon pathway involves binding to cells through specific receptors, activating molecular pathways, and up-regulating hundreds of ISGs. There are more than approximately 62 functionally validated ISGs, such as interferon-induced proteins with tetratricopeptide repeats (IFITs), protein kinase R (PKR), myxovirus resistance protein A (MxA), 2′-5′-oligoadenylate synthetase (OAS), viperin, ISG15 ubiquitin-like modifier (ISG15), and tudor domain-containing protein 7 (TDRD7), that function as antiviral genes as compared to the hundreds identified in mammalian studies [54,55]. ISG proteins disrupt viral replication, activate immune cells, and ensure that infected cells are killed [52,56,57,58]. Viral mimics, in turn, are proteins encoded by some pathogens that simulate or mimic elements of the host immune system, thereby enabling viruses to avoid or evade the host immune response. By mimicking cytokines, chemokines, or their receptors, these proteins allow viruses to disrupt signaling, inhibit immune responses, and persist within the host [59,60,61]. Viruses can also facilitate the generation of host-similar interferon proteins to circumvent the host antiviral response and survive in the infected host [62]. It is also through the IFN–viral mimic interaction that the host–pathogen relationship is understood, as in the eternal struggle. The evolutionary nature of the contact interplay between the host and the pathogen is one of the relevant aspects of the area of study. Also, in recent studies, viral imitators were identified in association with *Xenopus* pathogens that had not been studied previously [19]. Ranaviruses encode viral proteins that modulate host immune responses by specifically impairing Type I interferon signaling. The viral proteins act similarly to normal immune molecules that recognize pathogens. This allows the virus to escape detection, remaining in its host for extended periods. The way that ranaviruses can alter the pathways of cytokines in hosts shows that mimicry in viruses is important in the evolution of such diseases.

## 2. The IFN System in *Xenopus*

### 2.1. Discovery and Classification of Xenopus IFN

Early functional studies on *Xenopus* immune responses hinted at the presence of IFN-like activity, but molecular confirmation remained elusive until the advent of genomic and transcriptomic analyses [6]. The identification of IFN genes in *Xenopus* marked a pivotal advance in comparative immunology, revealing both evolutionary conservation and lineage-specific innovations within the amphibian IFN system.

Historically, *Xenopus* IFNs were grouped into three classes—Type I, Type II, and Type III—paralleling the mammalian nomenclature. However, as our understanding of amphibian immune complexity has deepened, this framework has been formally updated. It is now recognized that most vertebrates, including amphibians, possess four main IFN classes: Type I, II, III, and the newly designated Type IV, each associated with distinct receptors and effector functions [51,63]. Notably, *X. laevis* retains a functional *IFN-IV* gene, whereas in other *Xenopus* species, this sequence exists only as a pseudogene, underscoring a species-specific variation in immune gene repertoires within the genus [21,64].

Among these classes, Type I IFNs remain the best characterized. They induce early antiviral responses by signaling neighboring cells to upregulate protective effectors such as Mx proteins, PKR, and two OAS isoforms [65]. A recent comprehensive survey has revealed an unexpectedly expanded and complex Type I multigene family in *Xenopus*, comprising 42 genes in *X. laevis* and 51 in *X. tropicalis*. This family includes both intron-spanning and intronless members, a feature absent in mammals. Moreover, phylogenetic analyses cannot confidently subdivide these genes into distinct alpha or beta subtypes, as is done for mammalian Type I IFNs, highlighting a fundamental divergence in the organization and evolution of this family between amphibians and mammals [21]. Functionally, *Xenopus* Type I IFNs appear broadly antiviral, possibly reflecting adaptations to pathogen-rich aquatic and terrestrial environments. They are expressed across a wide range of cell types, in contrast to the more restricted expression patterns seen in mammals.

Type II IFN in *Xenopus* is represented by IFN-γ, which in mammals is produced principally by CD4^+^ and CD8^+^ T cells, innate-like T cells, and natural killer (NK) cells and is critical for macrophage activation and antigen presentation. In amphibians, however, IFN-γ responses have thus far been examined only at the tissue or whole-organ level. Elevated IFN-γ transcript levels have been observed in the spleen, kidneys, and liver of adult *X. laevis* during infections with FV3 or mycobacteria [66,67]. Notably, the gene for IFN-α7—a cytokine that plays crucial roles in adaptive immunity in higher vertebrates—is absent in *Xenopus*, suggesting that other pathways (e.g., expanded Type I IFN members or alternative cytokines) compensate for this in amphibian immune regulation.

Type III IFNs (IFN-λs) bind to the IFN-λ receptor and are primarily associated with mucosal immunity, protecting barrier surfaces such as the skin, lungs, and gastrointestinal tract. In *Xenopus*, IFN-λs are essential for defense against airborne and waterborne pathogens, consistent with the amphibian’s dual environmental exposure. Type III IFN has also been shown to play a key role in antiviral defense against ranavirus in *X. laevis* [68]. Unlike Type I IFNs, Type III expression is largely restricted to epithelial cells, aligning with its specialized role at mucosal surfaces [65].

Type IV IFN represents the most recently identified class. While its functional characterization in *Xenopus* is still emerging, the presence of a functional gene in *X. laevis*—versus a pseudogene in other species—points to ongoing evolutionary diversification within the genus [21,64].

Beyond class-level distinctions, developmental and evolutionary dimensions add further complexity. The dynamics of Type I and Type III IFN responses differ significantly between larval (tadpole) and adult stages [69]. Additionally, the amphibian lineage, including *Xenopus*, uniquely possesses both intron-containing and intronless IFN genes [20,70]. This genetic configuration likely facilitates greater diversity through alternative splicing and variant generation—a feature not observed in mammals—and may drive the expansion and diversification of IFN receptors, regulatory elements, and IFN-stimulated genes [21]. As such, *Xenopus* offers an invaluable model for investigating how IFN gene diversity evolves in vertebrates. Recent phylogenetic analyses of Type I IFN genes in *X. tropicalis* further suggest a divergence into terrestrial and aquatic branches, reflecting ecological pressures that have shaped the amphibian IFN system [7].

### 2.2. Intronless IFNs in Xenopus Immune Evolution

The evolution of interferons (IFNs) in amphibians represents a pivotal chapter in the history of this immune gene family, marked by the significant emergence of intronless (or single-exon) gene variants. Recent research has unveiled the surprising origin and expansion of these intronless IFN genes within amphibians [65,71]. For instance, each *Xenopus* species possesses approximately 50 predicted IFN genes, with a remarkable 24 to 37 of them being intronless [65]. This discovery revises the long-held model of IFN evolution, which previously placed the appearance of intronless IFNs in reptiles [65]. The unique coexistence of both intron-containing and intronless IFN genes in amphibians demonstrates that this pivotal evolutionary step actually occurred in amphibians or potentially even earlier in fish [65,71].

Furthermore, the expansion of these antiviral IFN genes, particularly the intronless forms, appears to be a species-specific process rather than one shared across all amphibian lineages. Evidence suggests that the intronless IFN genes in different amphibian species are primarily paralogs (genes related by duplication) rather than orthologs (genes related by speciation), and this relationship extends to the intronless IFNs found in amniotes [65,71,72]. Collectively, studies have confirmed that amphibians uniquely harbor both intron-containing and intronless forms within both the Type I and Type III IFN families [65,71,72,73]. These findings provide strong support for the idea that the retroposition mechanism, long considered the driving force behind intronless IFN evolution in higher vertebrates, actually originated with amphibians (or earlier) and not with reptiles as was previously thought [65,71,74,75,76].

The species-independent and parallel expansion of intronless Type I IFNs across different amphibian lineages points to a distinctive and critical role for this animal class in the broader narrative of IFN evolution. It is increasingly evident that this evolutionary innovation provided a significant adaptive benefit. The emergence of these intronless genes may have been crucial for enabling efficient gene expression and mounting a rapid and effective response against both pathogenic challenges and developmental pressures, ultimately facilitating the vertebrate transition to terrestrial life [20,65,71,77].

### 2.3. Molecular Mechanisms of IFN-Mediated Antiviral Immunity in Amphibian Model

**Figure 2:**
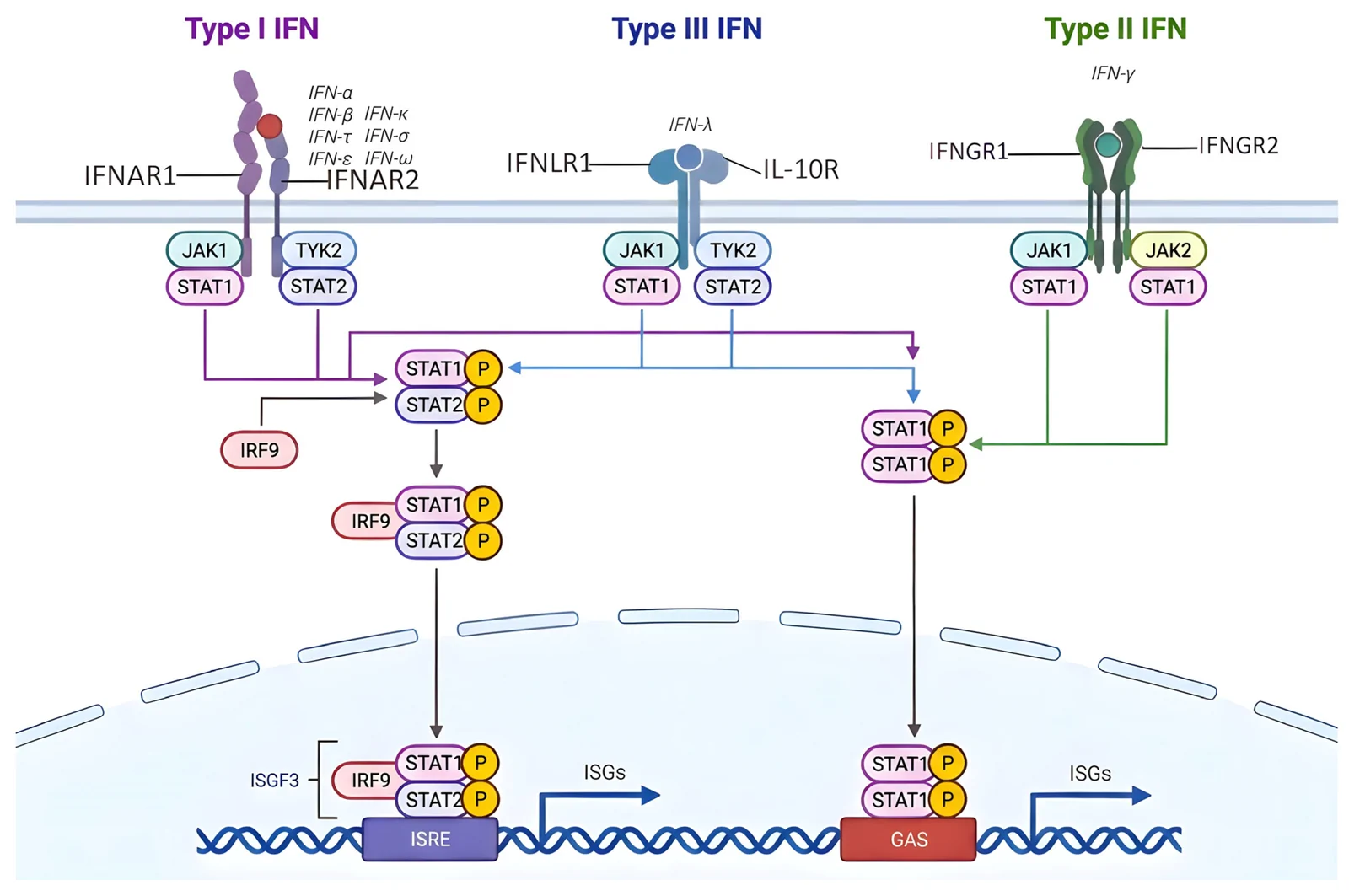
Mechanisms of IFN signaling in *Xenopus*: Upon viral infection, pattern recognition receptors (PRRs) detect pathogen-associated molecular patterns (PAMPs), triggering the production of Type I, II, and III IFNs. Type I and III IFNs engage their respective heterodimeric receptor complexes—IFNAR1/IFNAR2 or IFNLR1/IL10Rβ—while Type II IFN binds to IFNGR1/IFNGR2. Receptor dimerization activates the associated Janus kinases TYK2 and JAK1, leading to phosphorylation of STAT1 and STAT2. For Type I/III signaling, phosphorylated STAT1-STAT2 heterodimers associated with IRF9 to form ISGF3, which translocates to the nucleus and binds ISREs. In contrast, Type II IFN signaling drives STAT1 and STAT3 phosphorylation, forming heterodimers that bind GAS elements. The STAT1-STAT1 homodimer (often called GAF, or gamma-interferon activation factor) forms when interferon-gamma (IFN-γ) binds its cell-surface receptor. Both ISGF3 and IFN-γ activation factor (GAF) induce the transcription of hundreds of interferon-stimulated genes (ISGs), including key antiviral effectors such as myxovirus resistance (Mx), protein kinase R (PKR), and viperin, establishing a cellular antiviral state. The diagram also highlights cross-talk with NF-κB signaling and the differential expression of IFN genes in *Xenopus* tissues, underscoring the utility of this amphibian model for studying the evolution of vertebrate antiviral immunity. *Abbreviations*: IFNAR1, interferon alpha/beta receptor subunit 1; IFNAR2, interferon alpha/beta receptor subunit 2; IFNGR1, interferon gamma receptor 1; IFNGR2, interferon gamma receptor 2; IFNLR1, interferon lambda receptor 1; IL10Rβ, interleukin-10 receptor beta; JAK1, Janus kinase 1; TYK2, tyrosine kinase 2; STAT, signal transducer and activator of transcription; IRF9, interferon regulatory factor 9; ISGs, interferon-stimulated genes; ISGF3, interferon-stimulated gene factor 3; ISRE, interferon-stimulated response element; GAS, gamma-activated sequence; NF-κB, nuclear factor kappa-light-chain-enhancer of activated B cells. This figure was generated using the FigureLabs AI platform, version 2026 (https://chat.figurelabs.ai; accessed 29 May 2026) based on the authors’ original concept and instructions; the final image was reviewed and edited by the authors.

The interaction of IFN with its receptors triggers signal transducers and STATS that are critical to the amphibian immunological and antiviral functions (Figure 2). JAKs have been shown to mediate the activation of the STAT proteins in cross-species [78]. According to this work, the JAK-STAT pathway is believed to be conserved in vertebrates, with its roots dating back to ancient times. MX1, ISG15, and PKR are considered the primary interferon-stimulated genes in frogs that inhibit viral replication in various ways. For example, MX1 interferes with the assembly of the viral shell, and PKR restricts the creation of viral proteins [51]. PKR, another key interferon-stimulated gene, prevents viral protein creation by phosphorylating eukaryotic translation initiation factor 2 subunit alpha (eIF2α). This stops protein translation in the infected cell, thereby limiting the virus’s spread [79,80,81,82]. Also, ISG15 changes viral and host proteins to improve the antiviral immune response. The changes usually lead to the degradation of viral proteins.

Recent years’ studies have discovered various ISGs in *Xenopus*. These include viperin and the interferon-induced proteins with tetratricopeptide repeats (IFITs). They have antiviral functions [83]. Viperin stops viruses from making RNA, while IFIT can prevent viruses from changing RNA to produce lots of virus particles, blocking the viral life cycle. The mentioned ISGs enable *Xenopus* to mount a robust immune response against a wide range of viruses. Additionally, the significance of epigenetic regulation in IFN signaling is increasingly recognized. Methylation of DNA and histone modifications play a role in the modulation of the IFN response [7].

### 2.4. Evolutionary Conservation of IFN Pathways

Studies of evolution have suggested that IFN pathways in vertebrates are more functionally conserved than previously thought. An example is the JAK-STAT pathway found in fish, amphibians, and mammals. The existence of the JAK-STAT pathway implies an ancient origin in vertebrate evolution; it has been conserved because of its functions in immune responses. All these organisms have a JAK-STAT pathway in which IFNs bind their receptors on the cell surface, triggering JAKs, which in turn phosphorylate signal transducers and activators of transcription (STATs). These phosphorylated STATs have since moved to the nucleus, where they activate ISGs, which are vital for antiviral/immune activities, to be transcribed [20,51]. This conserved IFN signaling pathway in vertebrates indicates that the JAK-STAT pathway is an ancient immune signaling pathway that evolution has manipulated to ensure proper protection of organisms against infection. The same signaling cascade was also found to have been maintained in birds, amphibians, reptiles, and mammals because of its functional role in vertebrate immunity [84,85,86]. In this way, the host organisms would survive and fit in different niches.

*Xenopus* shares the IFN signaling pathway with the other vertebrates. It has also been demonstrated that *Xenopus* IFNs have developed gene duplications and lineage-based innovations. These gene replications produce different subtypes of IFN. Such subtypes can then serve specific functions in defense against viruses and bacteria and in the destruction of the environment during immune responses. These adaptations are believed to have helped *Xenopus* move into different habitats, such as water and land [20,45,51,87]. The gene duplications seen in *Xenopus* are not just confined to IFN family members but also other immune-related genes, which further diversify the amphibian immune system. *Xenopus* has IFN genes that underwent expansion, with species-specific adaptations, giving them the ability to ward off different pathogens [6,20,21]. Gene duplication may have played a significant role in aiding immune adaptation of novel IFN subtypes in *Xenopus*. Also, discovering similar regulatory components in IFN genes has shed light upon the evolutionary conservation of IFN pathways [7,20,21,88].

## 3. Viral Mimics in *Xenopus*

### 3.1. Definition and Examples of Viral Mimics

Viral mimics are proteins that a virus encodes that mimic host immune components to evade and manipulate the host immune response [60]. Viral mimics have been recognized in a number of DNA viruses in *Xenopus*, especially members of the Iridoviridae family, including ranaviruses and other iridoviruses [89,90,91,92]. Viruses are found to pose a serious threat to amphibians worldwide. Further, they have evolved many counter-host defenses. Ranaviruses can produce proteins with similarities to host cytokines that assist the virus in evading the host. Such identification of ISG-targeting viral mimics is part of the emerging trend in virus evolution, whereby pathogens evolve increasingly complex means to manipulate host immunity [93].

### 3.2. Mechanisms of Viral Mimicry

**Figure 3:**
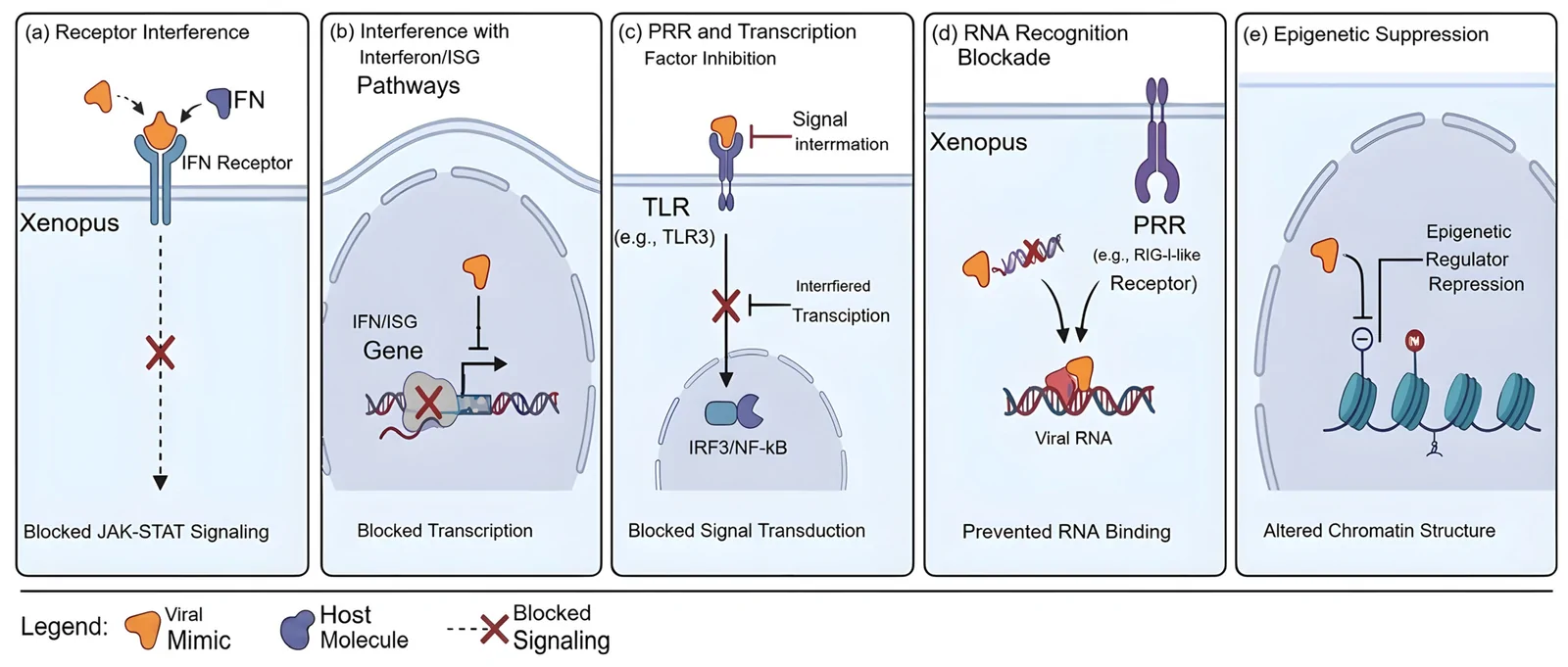
Mechanism of viral mimicry in *Xenopus*: This illustration depicts a mechanistic overview of how viral proteins mimic host immune components to subvert signaling pathways. (**a**) Viral mimics resemble host cytokines binding to IFN receptors to block signaling, (**b**) inhibiting interferon and ISG production, (**c**) interfering with pattern recognition receptors and key transcription factors (IRF3, NF-κB), (**d**) blocking viral RNA recognition, and (**e**) repressing host epigenetic regulators. The consequences are shown as weakened antiviral responses in *Xenopus* cells and how viral mimicry enables immune evasion and persistence in the host. *Abbreviations*: JAK-STAT, Janus kinase-signal transducer and activator of transcription; IRF3, interferon regulatory factor 3; ISG, interferon-stimulated gene; NF-κB, nuclear factor kappa-light-chain-enhancer of activated B cells; TLR, toll-like receptor; PRR; pattern recognition receptor; RIG-I, retinoic acid-inducible gene I. This figure was generated using the FigureLabs AI platform, version 2026 (https://chat.figurelabs.ai; accessed 29 May 2026) based on the authors’ original concept and instructions; the final image was reviewed and edited by the authors.

Viruses can evade their host’s immune response by hijacking components of the immune system [94,95,96]. Viruses replicate inside host cells. These tactics facilitate not only persistence within the host but also enable viruses to exploit the host’s immune system to their advantage. For instance, some mimics bind to IFN receptors and block the activation of downstream signaling pathways. Others inhibit the production of IFNs or ISGs, hence silencing the host antiviral response [51,81]. These illustrate the advanced mechanisms developed by pathogens to evade immune detection. Further, some viral mimics interfere with the host ability to recognize viral RNA, further enhancing immune evasion. By interfering with the synthesis or function of these key antiviral molecules, viruses manage to silence antiviral machinery effectively. It is one of the signaling pathways used to block the activities of pattern recognition receptors (PRRs) or the activities of the essential transcription factors, such as interferon regulatory factor 3 (IRF3) and nuclear factor kappa-light-chain-enhancer of activated B cells (NF-kB), which are vital in the production of IFN (Figure 3). This results in a weakening of the host’s immune system, making it difficult for the host to combat the virus; thus, the pathogen can easily overcome the host’s immune defenses [51,97,98].

Recent studies have uncovered novel mechanisms by which viruses that infect amphibians evade or manipulate the host innate immune system, particularly through the use of viral mimicry strategies. One of them is to repress host epigenetic regulators [99]. This research can empower us to discover the many ways the virus can elude the host defense system, a process that is known as viral mimicry. Viruses have a way of beating the immune system. To begin with, they can play around with receptor signaling. Secondly, they can disrupt interferon production [69,93]. They can also interfere with RNA recognition and even tamper with host epigenetics [95,100]. These mechanisms will help understand the evolution of host–pathogen interactions in other animal models, such as *Xenopus* (Figure 3). This demonstrates how viruses evade the host immune system and how the host immune system adapts in response to viral manipulation [90,97,101,102,103]. Accordingly, viral mimics have developed various strategies to neutralize immune responses in cells.

### 3.3. Evolutionary Implications of Viral Mimicry

The persistent presence of viral mimics in host–pathogen interactions acts not merely as a challenge to be overcome but as a powerful evolutionary catalyst that has fundamentally shaped the architecture of vertebrate immunity. By forcing host immune genes to continually diversify under selective pressure, these mimics drive a long-term process of adaptation that leaves indelible marks on the genome, which can be traced through patterns of positive selection and gene family expansion [89,91]. This viral replication leads to the diversification of immune genes. This interactive relationship helped develop the vertebrate immune system to counter viral infections more effectively. In most cases, vertebrates are effective against viruses. The study of viral mimics in *Xenopus* may provide insights into evolutionary processes, as the immune systems of this group of animals are less well researched than those of mammals. Nonetheless, it has a similar evolutionary capability to react to viruses. Research has been put forward to suggest that positive selection and gene duplication play a great role in immune response [7]. Among the processes, there is also gene duplication, that is, the host can generate a good number of copies of the immune-related genes, which can develop to react to different viruses [7]. It gives the host the ability to detect and destroy a great variety of pathogens, particularly those that employ viral mimicry strategies by mutation or dual copying of immune genes. Cloning of key reproductive genes made amphibians more armed against pathogens like ranaviruses that, regardless of their virulence, have always relied on viral mimics in order to destroy the host immune system. Similar patterns in the role of positive selection in the immune response have been observed in *Xenopus*. Positive selection can favor mutant forms of genes that give a selective advantage against pathogens [44,104]. This drives the evolution of the immune response component. The mutated component can recognize and neutralize a viral mimic more effectively. An example is showing how the host antiviral response has actively evolved under selective pressure, resulting in the evolution of receptors and signaling molecules that directly detect viral proteins and respond to them. Viruses continually change, indicating that they continue to adapt to evade immunity. It demonstrates that both the development of viruses and the immune system are interconnected.

All these findings pertain to the critical role of viral mimics in the coevolution of hosts and pathogens. Viruses exert selective pressure on their hosts’ immune systems, driving immune gene diversification through positive selection and gene duplication, which in turn shapes the host’s antiviral repertoire. The use of *Xenopus* models provides insights into these evolutionary dynamics, particularly in a non-mammalian vertebrate system, illustrating that the arms race between hosts and viruses is ongoing and context-dependent—where hosts evolve new recognition mechanisms, viruses concurrently adapt to evade them. This reciprocal process, rather than conferring universal protection against ‘more advanced’ viruses, highlights the continuous and specialized nature of host–pathogen coevolution.

## 4. Co-Evolution of *Xenopus* IFN and Viral Mimics

### 4.1. Arms Race Dynamics

The co-evolution of the *Xenopus* interferon system and its viral mimics exemplifies a dynamic, stepwise arms race, wherein each host innovation in immune defense is met with a reciprocal viral countermeasure aimed at subverting that very response. Unlike static adaptations, this continuous cycle of attack and defense drives the rapid turnover of immune genes and fosters the emergence of novel evasion tactics, such as the viral suppression of host epigenetic regulators. Investigating this specific antagonistic interplay in *Xenopus* provides a tractable model to dissect the immediate, reciprocal selection pressures that fuel the constant evolutionary struggle between a vertebrate host and its pathogens. Vertebrates are evolving components of immune genes that contribute to diversification and help develop complex immune systems [6]. The expansion of IFN genes in *Xenopus* has also been influenced by the need to resist viral mimics, suggesting that gene duplications are involved in the adaptation of the immune system [51]. New methods of imitating their hosts have been discovered in recent studies of viruses. For example, it can suppress host epigenetic regulators, providing a new perspective on the evolution of host–pathogen interactions [99]. An example of the functions of these viral mimics is to suppress host epigenetic regulators, including DNA methyltransferases, that suppress specific immune genes [105]. This viral trick demonstrates that certain pathogens are becoming increasingly sophisticated at interfering with the host’s immune system. It is necessary not only to research the various viruses but also their cross-species interaction. The combination of host immune dysfunction and viral pathogens is a continuously changing process. Therefore, *Xenopus* provides a good model for research on this. The core immune evolutionary mechanisms in vertebrates are being studied through *Xenopus* viral mimicry immune responses [90]. Learning how some vertebrates, e.g., frogs, develop various immune receptors can help develop new methods to counter infection. It may also be useful in opposing the threats that influence the evolution and dynamics of interactions between hosts and pathogens. The *Xenopus* studies still provide insights into host immune defense and pathogen evasion mechanisms through evolutionary immunology [6,26].

### 4.2. Gene Duplication and Diversification

A fundamental mechanism underpinning the adaptability of the *Xenopus* interferon system is the extensive gene duplication and subsequent functional diversification that has generated a diverse repertoire of IFN isoforms. Rather than simply increasing gene copy number, these duplication events have provided the raw genetic material for lineage-specific adaptations, allowing the host to develop specialized antiviral responses against a broad spectrum of pathogens. The identification of novel IFN subtypes, such as IFN-ω in *X. tropicalis*, highlights how this process of genetic innovation directly translates into enhanced and more nuanced immune capabilities, offering a concrete example of how genome plasticity facilitates evolutionary resilience. This is how immunity was created through genes that repeated multiple times during evolution [51]. There are a large number of IFN gene copies in the *Xenopus* genome, which may have led to enhanced immune responses and lineage-specific adaptations. These variations enable living beings to feel and respond more easily to different organisms. The IFN genes have evolved in *Xenopus*. This development has helped them combat pathogens. An excellent example of this is the identification of new subtypes of IFN, i.e., IFN-ω in *X. tropicalis*. Recent studies have shown that the new subtypes exhibit antiviral activity, offering insight into how genetic variants and corresponding functional modifications may lead to the development of a more specialized immune response [99]. The identification of IFN-γ indicates that gene duplication can yield different immune factors, and possibly, such instances can drive the evolution of specialized antibody responses that benefit the host in counteracting invasion. In addition, the study is being conducted to learn how epigenetic alterations, such as histone modifications and DNA methylation, can affect IFN responses through the regulation of immune gene expression [7]. These adaptations influence the ability of the immune system-related genes (i.e., the IFN system) to become more dynamic and adaptive to changes. More than 100 viruses have been isolated from amphibians [106]; however, the IFN (Type I) systems of frogs and newts are currently being investigated to determine whether they are functional [103]. The *Xenopus* IFN system can serve as a model for studying the evolution of vertebrate immune systems [21,77]. Thus, the IFN system can help the dominant principles of immunity over time.

### 4.3. Selective Pressure on Immune Genes

*Xenopus* IFN and viral mimics have coevolved, exerting intense selective pressure on immune genes. Thus, DNA and RNA viruses have shaped the evolution of immune genes. The IFN gene families may have diversified mainly by positive selection (i.e., adaptive evolution), while many immune trade-offs have been maintained by balancing selection, for instance, the trade-off between defense and autoimmunity [20,72,89]. Over time, vertebrate immune systems have evolved under selective pressure from pathogens and the microbiota, driven by an arms race. Simultaneously, selection for variety has an important role in preserving trade-offs for the immune system, especially against pathogens and autoimmunity. In this case, the immune system needs to balance an efficient immune response with overactivation that can harm the host or lead to chronic inflammation [89]. The immune systems of vertebrates, including *Xenopus*, have arisen through the combined forces of positive and balancing selection, together with flexibility in response mechanisms. Studies have shown conserved regulatory elements within shared IFN genes; hence, this evolutionary conservation is important in shared IFN pathways [6,7,45]. The conserved elements show that even with diversification and specialization, a common core of immune responsiveness remains ubiquitous across vertebrates, which is important in defense against pathogens. Additionally, transcriptomic and proteomic tools are helping researchers study the genetic basis of immune adaptation and host–pathogen interactions. Also, they enable the study of bacteria’s capacity to adapt to adaptive and innate immunity [99]. Research not only provides insight into the genetic variation in immunity but also shows that the interaction between the host and pathogen is not static. Scientists are studying the molecular basis of these interactions to better understand how vertebrates’ immune systems evolved to handle a variety of rapidly mutating pathogens.

### 4.4. Evolutionary Dynamics of Viral Mimicry

The emergence of viral mimics influences the evolution of the hosts and pathogens. Viruses can elicit diverse immune responses by evoking selective stress and mimicking the host immune system [89]. The evolutionary development of vertebrate life has led to modifications of the immune system due to the organism-pathogen interactions, consequently defining immunity as such. These have led to the development of vertebrate immune systems worldwide. The study of viral mimics in *Xenopus* will help understand how the evolution of the host–pathogen relationship affects the process of immunogenesis. Recently, the immunological reactions to viral mimics are formed by gene duplication and positive selection [7]. For example, the expansion of the *Xenopus* IFN family in response to viral mimics suggests that gene duplication is a frequent process. Viral genes that encode hypothetical proteins have been found to contain conserved domains and are potentially able to interfere with host interferon signaling [104]. Through our recent annotation efforts, we predicted and identified several viral mimics and viral coding genes, including putative open reading frames (ORFs) (Orf19R, Orf27R, Orf41R, Orf59L) and novel open reading frames (Norf) (Norf13L, Norf42L, and Norf66L). Most of these viral mimics contain regions resembling one or two IRF domains (vIRFs) of various vertebrate IRF proteins, while two share similarity with Fibronectin type 3 (FN3) domains (vFN3) of IFN receptor subunits that form the Type III IFNs—specifically, the IFN-λ receptor 1 (IFNLR1) and interleukin-10 receptor beta (IL10RB) [104]. Of note, the temporal class of these viral mimics (except the Norfs) had been reported before our recent studies as unknown (UNK) or L in previous studies [107].

### 4.5. Mechanisms of Immune Adaptation

The evolution of *Xenopus* IFN and viral mimics is valuable for numerous adaptive immune mechanisms. The expansions of *Xenopus* IFN genes are lineage-specific and thus increase the host’s response [51]. New IFN types have been discovered in the species *X. tropicalis* using recent studies. One such example is the use of interferon omega (IFN-ω), an antiviral agent [99]. These novel viruses are due to the evolution of the host’s defense against the virus. Moreover, another investigation has identified epigenetic control of IFN responses. It is established that immune responses are regulated by histone modification and DNA methylation [7]. Unlike the stable immune systems of many species, the *Xenopus* IFN system exhibits constant innovation and the ability to identify a wide variety of viruses, as well as to rapidly change its gene expression in response to new viruses. Collectively, these findings illustrate a recurring principle in vertebrate immune evolution: the capacity to withstand persistent microbial challenges is closely tied to the interplay between genetic diversification and epigenetic plasticity. Together, these mechanisms provide the functional flexibility necessary for long-term host survival in the face of rapidly changing infectious pressures.

### 4.6. Future Directions in Co-Evolution Research

Although such huge steps have been made, there are also some unanswered questions on how *Xenopus* IFN has co-evolved with viral mimics. This should be investigated further to describe the processes involved [51]. Mechanisms of the role played by epigenetic regulation in IFN signaling and the effect of contextual environmental forces on immune adaptation are not well understood. In addition, the definition of viral mimics and their activity will serve as a source of facts regarding how the relationships between hosts and pathogens develop. With the development of new technology, we create new jobs and better opportunities to upgrade our skills. Using high-throughput sequencing and comparative genomics, we can now more easily discover new immune-related genes and selection signatures in *Xenopus*; similarly, advances in high-throughput sequencing and CRISPR-Cas9 genome editing present new opportunities in *Xenopus* immunology [108]. Researchers can use these tools to understand the genetic basis of immune adaptation and host-pathogenic interactions [6]. We can create CRISPR/Cas9 knockout designs targeting IFN genes to elucidate function. Also, scientists will be able to study immune responses at the cellular level thanks to advances in single-cell RNA sequencing. In contrast to conventional RNA sequencing, which averages signals across many cells, single-cell RNA sequencing (scRNA-seq) captures the transcriptional heterogeneity in immune tissues. It identifies distinct cellular states, activation patterns, and rare cell types that can be beneficial in the defense against pathogens. Single-cell strategies can help elucidate how macrophages, dendritic cells, and lymphocytes differentially express IFN genes and respond to viruses within the context of IFN biology [109,110]. CRISPR/Cas9-mediated gene editing and single-cell transcriptomics together allow high-resolution mapping of immune cell heterogeneity and gene expression patterns, as well as generation of genetically engineered *Xenopus* models for other applications [20,30,95,111,112,113,114], thereby providing a complementary toolkit for unraveling the complexity of the *Xenopus* immune system and a more general understanding of vertebrate immune evolution.

## 5. Comparative Immunology: Insights from *Xenopus*

### 5.1. Evolutionary Conservation of IFN Pathways

The common pathways of the immune response have been known to be comparable across vertebrates. One of them is the JAK-STAT, which is found in fish, amphibians, and mammals, showing that it is a highly conserved pathway [51]. There are other *Xenopus* IFNs’ peculiarities, like specific-lineage adaptations. The results of recent studies have supported the idea that IFN genes have their own regulating elements and transcriptional activation of ISGs requires ISREs [7]. The conclusions suggest that the *Xenopus* evolutionary experimental model can be used to investigate the conservation of the immune system. Also, *X. tropicalis* has been spotted to possess certain new subtypes of interferons, and this has assisted in augmenting our knowledge of the interferon gene variations and the effects they would have on antiviral defense.

### 5.2. Unique Features of Xenopus IFN System

The gene duplication and functional specialization observed in *Xenopus* represent key features of its IFN system [45]. These characteristics are essential for understanding the evolution of the immune system and the development of host–pathogen interactions [6]. This has been due to their lineage, which has contributed to the growth of the *Xenopus* IFN genes, which has enhanced the rampant ability to act against other pathogenic organisms. New IFN subtypes were identified in *X. tropicalis*, like IFN-ω, recently. In addition to it, an interesting direction of research is associated with epigenetic control of IFN responses; research works were performed in the context of histone modification and DNA methylation control of immune responses [7]. These results indicate the nature of the *Xenopus* IFN system and its ease in evolving. Once we learn the role of molecular machinery in the speciation of IFN genes and epigenetic regulation, we will be able to develop more appropriate means to counter infectious diseases.

## 6. Experimental Evidence and Case Studies

### 6.1. Studies on Xenopus IFN Responses

Scientists have shown that IFNs are vital for fighting viruses in *Xenopus* [20]. Ranavirus infections trigger an excessive IFN response, which inhibits viral multiplication [51]. The work provided excellent insight into the laws governing IFN functions and viral defense via ISGs. More recent data reports additional ISGs in *Xenopus* required for antiviral defense, including viperin and IFITs. In addition, the development of CRISPR/Cas9 technology enabled the experimental silencing of IFN genes in *Xenopus*, providing another study of their role in the immune response [30]. Experiments by scientists that have interfered with IFN receptors have demonstrated that IFN receptors are significant in fighting viruses and regulating the immune response.

### 6.2. Viral Mimics in Xenopus Pathogens

Viral mimics are viral proteins or nucleic acid structures that structurally or functionally mimic host immune molecules, thereby subverting or evading host immune responses. In *Xenopus*, different viral mimics have been detected, including ranaviruses and iridoviruses. All these mimics block key IFN pathway endpoints, thereby evading host immunity [89]. An example is ranaviruses, which produce cytokine-like proteins in the host and, consequently, harm the immune system [19]. Recent research revealed more viral mimics found in *Xenopus* pathogens. This demonstrates that viruses have numerous ways to elicit the immune system.

Of interest to scientists is the interaction between the *Xenopus* protein domains directly mediating IFN signaling and the viral gene. They include those that are found in IFN receptors and IRFs. Viruses can form false imitations, or homologs, of host proteins to evade vital antibodies. By identifying and mapping the unique domains of architecture of *Xenopus* IFN receptors systematically, the researchers can produce a detailed molecular portrait of the defensive machinery of the host, that is, fibronectin Type III domains involved in ligand binding, DNA-binding domains (DBD), and association domains with the IRF in their own right, i.e., IRF association domains (IAD). It serves as a guide for bioinformatics screens of viral genomes: the identification of genes whose products encode related domains with little overall sequence homology is a strong signature of a viral counterstrategy to interfere with IFN-mediated immunity. Focusing on the domain enables the use of the *Xenopus* model, which occupies a unique evolutionary position and helps study the ancient conflict between viruses and the vertebrate immune system [20].

### 6.3. Evolutionary Studies Using Xenopus

By comparing genomes and analyzing the evolution of *Xenopus* IFN genes, researchers gained valuable insights into the evolution of these genes. The research shows how the host and pathogen interact. Several studies of hybrids also demonstrate that the host–pathogen arms race shapes interactions in these hybrids [51]. For instance, expanding IFN genes of *Xenopus* combat viral mimics (Table 2). Thus, gene duplication can assist in immune adaptation. Scholars have discovered conserved regulatory elements in the IFN genes, revealing the evolutionary conservation of IFN pathways [7].

## 7. Implications for Evolutionary Biology

### 7.1. Insights into Host–Pathogen Co-Evolution

The evolutionary arms race between *Xenopus* and its pathogens extends beyond classical gene duplication events, revealing a more nuanced conflict that operates at the level of chromatin regulation. While genetic expansion of immune loci represents one layer of adaptation, recent evidence highlights that viruses employ sophisticated counter-strategies that directly interfere with host epigenetic machinery. For instance, viral proteins have been shown to mimic host histone motifs or recruit chromatin-modifying enzymes to suppress antiviral gene expression without altering the underlying DNA sequence [125]. This form of molecular mimicry can block the activity of host epigenetic regulators, such as histone methyltransferases and acetyltransferases, thereby silencing interferon-stimulated genes and other defense effectors [125].

This discovery shifts the co-evolutionary perspective from a purely sequence-based conflict to one that also involves transcriptional accessibility and nuclear architecture. Consequently, the host response must evolve not only to recognize and neutralize viral proteins but also to safeguard its epigenetic regulators from subversion. This imposes selective pressure on non-coding regulatory elements, histone variant usage, and the expression of epigenetic “sensor” proteins that can distinguish self from foreign modulators [126]. The interplay between viral mimicry and host epigenetic defense thus constitutes a rapidly evolving front in vertebrate immunity—one that is largely invisible to classical phylogenetic analyses that focus solely on coding sequence divergence.

In this context, *Xenopus* offers a distinctive experimental platform. Its phylogenetic position at the boundary between fish and amniotes, combined with the tractability of its externally developing embryos and the availability of transgenic and CRISPR-Cas9 tools, allows researchers to functionally test how epigenetic perturbations affect pathogen susceptibility over evolutionary timescales [6,127]. Unlike mammalian systems, where functional redundancy among epigenetic factors often obscures individual contributions, *Xenopus* presents a comparatively streamlined epigenetic landscape. This enables direct inquiry into whether particular viral epigenetic mimics drive compensatory changes in host histone modification patterns and whether such changes are transgenerationally heritable—questions that are central to understanding the tempo and mode of co-evolutionary adaptation [126,128].

In summary, the emerging focus on epigenetic conflict reframes host–pathogen co-evolution as a multidimensional process, wherein the battleground extends from DNA sequence to chromatin state. *Xenopus* stands as a powerful model to dissect these interactions, offering mechanistic insights that are complementary to, and not redundant with, classical genetic studies of immune gene expansion.

### 7.2. Applications in Comparative Immunology

The *Xenopus* model can be used in a comparative study of the immunology of fish and mammals. Because *Xenopus* is phylogenetically related to these groups, it enables the discovery of the origins of critical immune elements in vertebrates and their divergence. *Xenopus* research contributes to the study of the origin and evolution of vertebrate immunity [51]. Increased expression of IFN genes in *Xenopus*, e.g., led to lineage-specific adaptations and enhanced the host’s fitness against diverse pathogens. Recently, new IFN subtypes were identified in *X. tropicalis*, and it was shown that gene duplication is adapted to the immune response [99]. Recent studies of immune genes in *Xenopus* have also now been enabled by the advent of CRISPR/Cas9 technology [30].

### 7.3. Potential Applications and Evolutionary Insights of Xenopus IFNs

The IFN system of the African clawed frog, *X. laevis*, offers a uniquely powerful lens through which to investigate both the evolution of vertebrate immunity and the developmental dynamics of antiviral defense. As one of the most basal jawed vertebrates possessing both Type I and Type III IFN cytokines, *Xenopus* occupies a critical phylogenetic position that allows researchers to distinguish evolutionarily conserved immune mechanisms from species-specific adaptations. Beyond this comparative advantage, the model reveals a striking functional division across life stages: vulnerable tadpoles mount a robust type III IFN (IFN-λ) response to combat pathogens like FV3, whereas adult frogs transition to a predominantly type I IFN-driven defense [68,69,70]. This developmental switch alters the tissue-specific expression of antiviral genes and provides a natural experimental system for understanding how young organisms manage severe viral threats while their immune systems are still maturing. Furthermore, the recent discovery of a novel Type IV IFN (IFN-υ) system in *Xenopus* adds another layer of complexity to the amphibian antiviral arsenal, offering a unique window into the diversification and sub-functionalization of IFN families across vertebrates [126,127]. This intricate network of distinct IFN types, each with potentially specialized functions and tissue specificities, positions *Xenopus* as an indispensable model for dissecting the fundamental mechanisms of IFN-mediated immunity and host–pathogen interactions.

The characterization of *Xenopus* IFNs carries substantial translational potential, as these amphibian proteins serve as natural blueprints for designing next-generation antiviral therapeutics. Studies have demonstrated that recombinant *Xenopus* Type I and Type III IFNs can elicit potent antiviral gene expression and significantly reduce FV3 replication both in cell lines and in vivo, even extending tadpole survival following lethal viral challenge [68,127]. This provides a valuable preclinical platform for testing recombinant IFN therapeutics and for understanding how pathogens evolve to subvert these responses. For instance, FV3 employs immune evasion strategies, such as impairing IFN-λ receptor expression, an effect that can be effectively overcome by exogenous recombinant IFN treatment. Moreover, the unique signaling properties of *Xenopus* IFNs help researchers map cross-species immune pathways, illuminating how mammalian systems evolved to handle intracellular pathogens and offering insights that could inform the design of broad-spectrum antivirals capable of combating emerging zoonotic viruses [68].

Beyond pathogen-specific applications, *Xenopus* IFN research has uncovered unexpected connections between endogenous elements and innate immunity. Intriguingly, studies have shown that endogenous retroviruses (ERVs) can activate the IFN axis in tadpoles, providing intrinsic antiviral protection that could inform novel prophylactic strategies [95]. This finding highlights how the amphibian model can reveal fundamental principles of immune regulation that are difficult to observe in mammalian systems. Additionally, the highly diversified IFN families of *Xenopus* serve as natural blueprints for creating versatile, synthetic antiviral drugs, while the developmental stage-specific IFN responses provide a unique system for studying the interplay between immune maturation and viral susceptibility [68,95]. Ultimately, by providing a platform to investigate IFN regulation, signaling, and function across both evolutionary time and developmental stages, *X. laevis* research not only enhances our fundamental understanding of immunology but also holds concrete promise for developing novel antiviral therapeutics and vaccines for both veterinary and human medicine [25,46].

### 7.4. Broader Evolutionary Questions

Research on *Xenopus* IFN and its interactions with viral mimics poses a broader challenge to the application of immune genes in speciation and adaptation. Immune system genes are expected to be under severe selection pressure because they interact with rapidly evolving pathogens, particularly those that elicit direct antiviral immune responses. Studies indicate that genes in the immune system play a role in shaping evolutionary trends [89]. Conserved regulatory elements in IFN genes have been reported, providing insight into IFN pathway conservation in broader evolutionary terms [7]. Based on these results, a balance is maintained between mutation, as indicated by gene duplications and positive selection, and the preservation of key regulatory processes and mechanisms required for functional expression [7]. Immune genes in non-mammalian model organisms, such as *Xenopus*, are more widely conserved.

## 8. Future Directions

### 8.1. Unresolved Questions in Xenopus Immunology

Although scientists have learned much about *Xenopus* immunology, many intriguing questions remain about *Xenopus* IFN, viral mimics, and the evolution of regulation. The identification of new IFN subtypes and the methods involved in their interaction with pathogens is really very useful information; however, it is very complicated. Future research should explain the mechanisms involved in these processes [51]. For example, the influence of epigenetic control on IFN signaling and the effect of environmental factors on immune adaptation are largely unknown. The role of these changes in controlling IFN gene expression during development, infection, or environmental stress could provide a valuable clue to immune plasticity and memory [19,89]. One aspect that can be studied later is the influence of environmental conditions on the immune system, such as temperature, pollutants, and exposure to microorganisms, which may affect its function. Amphibians are particularly susceptible to environmental degradation; hence, they are ideal subjects for research on immune evolution and variability under ecological pressure. Studies could help elucidate how immune systems adapt to fluctuating environments and how such adaptations may affect disease susceptibility. Furthermore, finding new viral lookalikes and understanding how they function will help reveal how the battle between invading viruses and their hosts has changed and evolved. In the end, filling these gaps would help us trace the evolution of immunity in vertebrates and develop solutions for disease control, conservation biology, and drug development.

### 8.2. Applications in Medicine and Biotechnology

Knowledge from *Xenopus* immunology has practical medical and biotech applications. *Xenopus* is a good model organism that provides insights into immune system evolution and function, given its comparative invertebrate and mammalian nature. Viral mimicry is a potentially fruitful field of translational research, i.e., how viruses evade or subvert host immune responses. Recent antiviral medications and vaccines can emerge from the information about viral mimicry [89]. Recent investigations on *X. tropicalis* have identified new IFN subtypes that may be used in biotechnology and medicine. New species of interferons found in the *X. tropicalis* frog can have novel classes of antiviral parameters, and might hence establish new categories of IFN-based therapy. The new types that are interferon-based can provide excellent antiviral drugs against diseases like autoimmune conditions, cancers, and viruses [99]. It will not only be known by the manner in which those IFNs actually work in a non-mammalian immune system but also be much easier to evolve and to allow us to regulate this evolvability in order to treat diseases. The availability of a large number of genetic and transcriptomic tools for *Xenopus* has expanded recently, making this organism increasingly accessible for biomedical research [128]. This model, besides enabling the study of immune system development, has provided platforms for discovering molecules and pathways that would otherwise have practical applications in medicine and biotech.

## 9. Conclusions

The *Xenopus* IFN complex is a vital subject under study, along with viral mimics, in which we have made splendid observations on the evolution of vertebrate immunity. In this review, the development of both hosts and pathogens is represented, and the replication of genes in the immune system is shown to be an evolutionary mechanism. *Xenopus* is a crucial intermediate between fish and mammalian immunology for discovering the origin and evolution of vertebrate immunity. A force contributing to an evolutionary arms race is gene duplication. It provides genetic data on novel functionality and enables the development of custom-designed immune adaptations within a few lineages. The information presented in our results provides insight into the development of the immune system over time and, therefore, into its possible real-life uses. We can understand the mechanism by which *Xenopus* IFNs counteract viral mimics to develop new antiviral drugs to optimize vaccines by conservative immune pathways. In future studies, researchers should examine the mechanisms underlying immune adaptation in vertebrates and their applications in biotechnology and medicine. Including what we find in biomedical contexts could help us develop therapeutic strategies that leverage the evolutionarily derived features of vertebrate immunity. In conclusion, *Xenopus* is a model of evolutionary significance and a vehicle for medicine and biotechnology.

## Figures and Tables

**Table 1:** Key areas of immunological research using *Xenopus*.

Research Area	Key Advantage of *Xenopus*	Specific Applications and Findings	References
Immune system development (ontogeny) evolution	External development free of maternal influence; accessibility of the thymus from the earliest stages.	Studies on the early commitment and fate of myeloid and lymphoid lineages. Surgical thymectomy at early stages generates T-cell-deficient animals to study T-cell function in vivo.	[6,25,26]
Disease and host–pathogen interactions	Relevance to both wildlife disease ecology and fundamental antiviral/antifungal immunity.	A key model for studying emerging amphibian pathogens like Frog Virus 3 (FV3) and the chytrid fungus *Batrachochytrium*, which is vital for conservation. It also helps elucidate the role of skin microbiomes in immune defense.	[41,42,43,44]
Immunogenetics and comparative immunology	The existence of species with different ploidy levels offers a unique model to study gene regulation at the genome level.	Analysis of immune gene families has revealed diversification, expansion, and contraction patterns, such as in nonclassical MHC class I genes and cytokine families.	[45,46]
Cancer and tumor immunity	MHC-defined strains and T-cell-deficient models allow for detailed analysis of antitumor immune responses.	Study of immune responses to tumors, facilitated by the availability of lymphoid tumor cell lines for in vivo and in vitro studies.	[25,47]
Regeneration, autoimmunity and immunotoxicology	Metamorphosis as a model for immune system remodeling and tolerance induction.	Studies on vascular and lymphatic regeneration in transparent tadpoles. The immune system’s reorganization at metamorphosis provides a unique setting to study self-tolerance. Also used to assess the impact of environmental toxins on immune development.	[48,49,50]

**Table 2:** Selected viral mimics in vertebrate pathogens and their mechanisms of action.

Viral Mimic	Pathogen	Target	Mechanism of Action	Evolutionary Implication	References
vIFN-γ	Ranavirus	Host IFN-γ receptor	Binds to IFN-γ receptor, blocking downstream signaling	Drives diversification of IFN receptors and signaling pathways	[115,116]
vIL-10	Iridovirus	Host IL-10 receptor	Mimics host IL-10, suppressing pro-inflammatory responses	Promotes immune tolerance and viral persistence	[117]
vISG15	Ranavirus	Host ISG15	Mimics ISG15, interfering with host antiviral responses	Drives the evolution of ISG15 and related ISGs	[101]
vJAK	Iridovirus	Host JAKs	Inhibits JAK-STAT signaling, blocking IFN responses	Promotes diversification of JAK-STAT pathway components	[115,118]
vSOCS	Ranavirus	Host JAK/STAT pathway components	Degrades or inhibits JAKs and signaling intermediates	Selects for redundancy and variation in cytokine signaling cellular genes (SOCS) box proteins	[119,120]
vIκB	Iridovirus	Host NF-κB pathway	Binds to NF-κB, preventing its nuclear translocation and activation of antiviral genes	Drives the evolution of alternative NF-κB activation pathways	[121]
vCD200	Ranavirus	Host CD200 receptor	Delivers an inhibitory signal to macrophages and dendritic cells	Promotes diversification of inhibitory receptor signaling	[122]
vGalectin	Iridovirus	Host cell surface glycans	Modulates cell adhesion and induces apoptosis in activated T-cells	Drives glycan diversity and immune cell evasion strategies	[123,124]

*Abbreviations*: vIFN-γ, viral interferon-gamma; vIL-10, viral interleukin-10; vISG15, viral interferon-stimulated gene 15; vJAK, viral Janus kinase; vSOCS, viral suppressors of cytokine signaling; vIκB, viral homolog of the cellular Iκ B (inhibitor of κ B) protein; vCD200, viral homologues of the cellular CD200 (cluster of differentiation 200) protein; vGalectin, viral galectin; NF-κB, Nuclear factor kappa-light-chain-enhancer of activated B; SOCS, suppressors of cytokine signaling; JAK-STAT, Janus kinase-signal transducer and activator of transcription.

## Data Availability

No new data were created or analyzed in this study. Data sharing is not applicable to this article.

## Abbreviations

The following abbreviations are used in this manuscript:

CD4^+^	Cluster of differentiation 4 positive
CD8^+^	Cluster of differentiation 8 positive
CRISPR-Cas9	Clustered regularly interspaced short palindromic repeats and CRISPR-associated protein 9
DBD	DNA-binding domain
eIF2α	Eukaryotic translation initiation factor 2 subunit alpha
FN3	Fibronectin type 3
FV3	Frog Virus 3
GAF	Gamma activation factor
GAS	Gamma-activated sequence
IAD	IRF association domain
IFIT	Interferon-induced proteins with tetratricopeptide repeat
IFN	Interferon
IFNAR	Interferon-alpha receptor
IFN-α7	Interferon alpha-7
IFN-γ	Interferon-gamma
IFN-λ	Interferon-lambda
IFN-ω	Interferon omega
IL10RB	Interleukin-10 receptor beta
IRF3	Interferon regulatory factor 3
IRF9	Interferon regulatory factor 9
ISG	Interferon-stimulated gene
ISGF3	Interferon-stimulated gene factor 3
ISRE	Interferon-stimulated response element
JAK	Janus kinases
MHC	Major histocompatibility complex
Mx	Myxovirus resistance
MxA	Myxovirus resistance protein A
NF-kB	Nuclear factor kappa-light-chain-enhancer of activated B cells
NK	Natural Killer
Norf	Novel open reading frame
OAS	Oligoadenylate synthetase
ORF	Open reading frame
PAMP	Pathogen-associated molecular pattern
PKR	Protein Kinase R
PRR	Pattern recognition receptors
RIG-I	Retinoic acid-inducible gene I
scRNA-seq	Single-cell RNA sequencing
STAT	Signal transducers and activators of transcription
TDRD7	Tudor domain-containing protein 7
TLR	Toll-like receptor
TYK2	Tyrosine kinase 2
vCD200	viral homologues of the cellular CD200 (cluster of differentiation 200) protein
vGalectin	viral galectin
vIFN-γ	viral interferon-gamma
vIL-10	viral interleukin-10
vISG15	viral interferon-stimulated gene 15
vIκB	viral homolog of the cellular Iκ B (inhibitor of κ B) protein
vJAK	viral Janus kinase
vSOCS	viral suppressors of cytokine signaling
